# KDM1 is a novel therapeutic target for the treatment of gliomas

**DOI:** 10.18632/oncotarget.725

**Published:** 2012-11-17

**Authors:** Gangadhara R. Sareddy, Binoj C. Nair, Samaya K. Krishnan, Vijay K. Gonugunta, Quan-guang Zhang, Takayoshi Suzuki, Naoki Miyata, Andrew J. Brenner, Darrell W. Brann, Ratna K. Vadlamudi

**Affiliations:** ^1^ The Department of Obstetrics and Gynecology, University of Texas Health Science Center at San Antonio, San Antonio TX; ^2^ Institute of Molecular Medicine and Genetics, Georgia Health Sciences University, Augusta, GA; ^3^ Graduate School of Medical Science, Kyoto Prefectural University of Medicine, 13 Taishogun Nishitakatsukasa-Cho, Kita-ku, Kyoto, Japan; ^4^ PRESTO, Japan Science and Technology Agency (JST), 4-1-8 Honcho Kawaguchi, Saitama, Japan; ^5^ Graduate School of Pharmaceutical Sciences, Nagoya City University, 3-1 Tanabe-dori, Mizuho-ku, Nagoya, Aichi, Japan; ^6^ Department of Hematology and Medical oncology, University of Texas Health Science Center at San Antonio, San Antonio TX; ^7^ Department of Cancer Therapy & Research Center, University of Texas Health Science Center at San Antonio, San Antonio TX

**Keywords:** KDM1, p53, Gliomas, GBM, epigenetics

## Abstract

Glioma development is a multistep process, involving alterations in genetic and epigenetic mechanisms. Understanding the mechanisms and enzymes that promote epigenetic changes in gliomas are urgently needed to identify novel therapeutic targets. We examined the role of histone demethylase KDM1 in glioma progression. KDM1 was overexpressed in gliomas and its expression positively correlated with histological malignancy. Knockdown of KDM1 expression or its pharmacological inhibition using pargyline or NCL-1 significantly reduced the proliferation of glioma cells. Inhibition of KDM1 promoted up regulation of the p53 target genes p21 and PUMA. Patient-derived primary GBM cells expressed high levels of KDM1 and pharmacological inhibition of KDM1 decreased their proliferation. Further, KDM1 inhibition reduced the expression of stemness markers CD133 and nestin in GBM cells. Mouse xenograft assays revealed that inhibition of KDM1 significantly reduced glioma xenograft tumor growth. Inhibition of KDM1 increased levels of H3K4-me2 and H3K9-Ac histone modifications, reduced H3K9-me2 modification and promoted expression of p53 target genes (p21 and PUMA), leading to apoptosis of glioma xenograft tumors. Our results suggest that KDM1 is overexpressed in gliomas and could be a potential therapeutic target for the treatment of gliomas.

## INTRODUCTION

Gliomas are the most malignant primary brain tumors and account for more than 70% of all primary brain tumors [1;2]. Current therapies result in a median survival of 12–15 months [3]. Therefore, understanding the mechanisms of glioma development and finding new therapeutic options is clinically significant. Glioma development is a multistep process that results from changes in genetic and epigenetic mechanisms [4;5]. Histone methylation is a dynamic process that has been associated with either transcriptional activation or repression [6] and emerging evidence suggests that alterations in histone methylation play a vital role in many neoplastic processes [7]. Unlike genetic alterations, epigenetic changes are reversible and thus, targeting epigenetic changes represents a promising therapeutic approach.

The lysine-specific demethylase-1 (KDM1; also known as LSD1, AOF2 or BHC110) gene encodes a flavin-dependent monoamine oxidase, which can demethylate mono- and dimethylated lysines, specifically histone H3, lysines 4, 9, 27 and 36 [8]. KDM1 is highly conserved in eukaryotes and required for many physiological functions [9]. Recent investigations found that KDM1 interacts with nuclear receptors and chromatin-modifying corepressor complexes such as the Co-Rest complex [10;11]. KDM1 also interacts with non-histone substrates including p53 [12] and pRb [13]. KDM1 is overexpressed in different cancer types, including neuroblastoma [14], prostate, breast, and colon cancers [15]. Even though these findings suggest a role of KDM1 in cancer progression, the significance of KDM1 in glioma progression remain elusive.

In this study, we examined the status of KDM1 in gliomas by using tumor tissue arrays and tested the therapeutic significance of pharmacological inhibition of the KDM1 axis in gliomas by using both *in vitro* and *in vivo* preclinical xenograft models. Our results demonstrate that deregulation of KDM1 expression occurs during glioma progression with highest expression in high-grade gliomas. Pharmacological inhibition of either KDM1 activity or knockdown of its expression via siRNA reduces the proliferation of established as well as patient-derived primary GBM cells. Mechanistic studies showed that KDM1 inhibitors promote apoptosis of glioma cells via activation of p53 pathway.

## RESULTS

### KDM1 is overexpressed in gliomas and its expression correlates with histological malignancy

**Figure 1:**
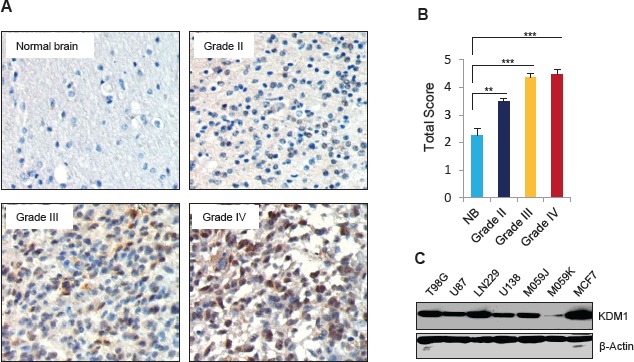
KDM1 expression is elevated in gliomas (A) Glioma tissue microarray containing control brain (n=16), as well as grade II (n=130), grade III (n=29) and grade IV (n=33) glioma specimens was subjected to immunohistochemical staining with the KDM1 antibody as described in the methods section. (B) Quantitation of total score in each grade was done as described in the methods section, bars, SEM. **, *p*< 0.05. ***, *p*< 0.001 (C) Whole cell lysates from various glioma cell lines were subjected to Western blot analysis, and blots were probed with KDM1 antibody. MCF7 cells were used as a positive control and β-actin served as internal control.

Recent evidence attributed an oncogenic role for KDM1 in various malignancies [19]. To determine the status of KDM1 in gliomas, we checked the expression of KDM1 using glioma tissue arrays that contain the different grades of gliomas as well as normal brain tissues and the intensity of staining was scored as described previously [18]. The representative staining for each grade is shown in Fig. 1A. KDM1 expression was significantly higher in gliomas than in normal brain tissues and positively correlated with histological malignancy (Fig. 1B). Western blot analysis of total lysates from glioma cell lines revealed that higher KDM1 expression in the majority of the tested glioma cell lines (Fig. 1C). These results suggest that KDM1 is highly expressed in gliomas.

### KDM1 inhibition reduces the glioma cell proliferation and colony formation

**Figure 2:**
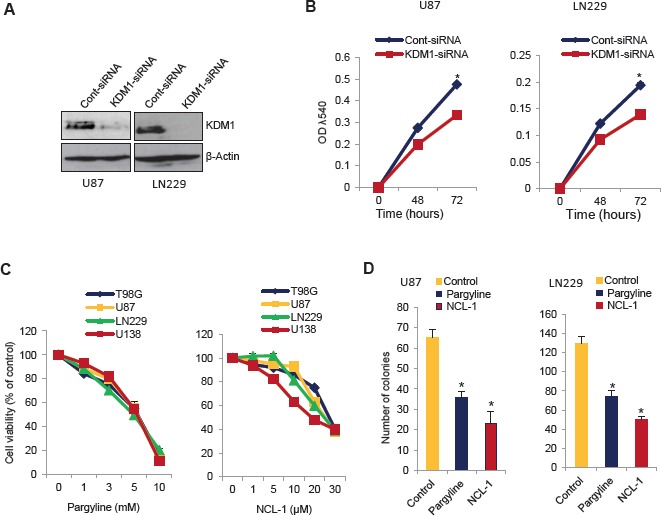
Inhibition of KDM1 expression or functions reduced the proliferation of glioma cell lines (A) U87 and LN229 glioma cells were transiently transfected with KDM1 siRNA or control siRNA using the oligofectamine transfection reagent. After 72 h, whole cell lysates were subjected to Western blot analysis using the KDM1 antibody. β-actin was used as a loading control. (B) U87 and LN229 cells transfected with KDM1 siRNA or control siRNA were seeded in 96-well plates (2 × 10^3^ cells/well) and the proliferation was determined at 48 h and 72 h by using the MTT assay. All data presented are the mean of 3 independent experiments with mean ± SEM. *, *p*< 0.05. (C) T98G, U87, LN229 and U138 glioma cells were treated with vehicle or varying concentration of pargyline or NCL-1 for 72 h, and proliferation was measured by using the MTT assay. (D) U87 and LN229 cells were seeded in 6-well plates and treated with vehicle, pargyline, or NCL-1 for 72 h and allowed to grow for additional 7 days. Colonies were stained with crystal violet and colonies that contain ≥50 cells were counted. All data presented are the mean of 3 independent experiments with mean ± SEM. *, *p*< 0.05, *t* test.

To examine the significance of KDM1 in glioma cells, we knocked down KDM1 expression by using the siRNA approach. U87 and LN229 cells were transiently transfected with KDM1-siRNA or control-siRNA. Western blot analysis showed that KDM1 expression was ~80% less in KDM1-siRNA than in control siRNA transfected cells (Fig 2A). Cell proliferation assays revealed that glioma cell proliferation in KDM1 knockdown cells was significantly less than that of control siRNA (Fig 2B). To further probe the function of KDM1, we used pharmacological inhibitors of KDM1 (pargyline and NCL-1) that are known to inhibit the KDM1 enzyme activity [20]. As shown in Fig. 2C, treatment of glioma cells with either pargyline or NCL-1 significantly reduced their proliferation in a dose-dependent manner. Further, clonogenic assays revealed that pargyline and NCL-1 significantly reduced the colony formation ability of glioma cells (Fig. 2D). These results suggest that inhibition of KDM1 has potential to impair the glioma cell proliferation.

### KDM1 inhibition modulates acetylation of p53 and activates its target gene expression p21 and PUMA

**Figure 3:**
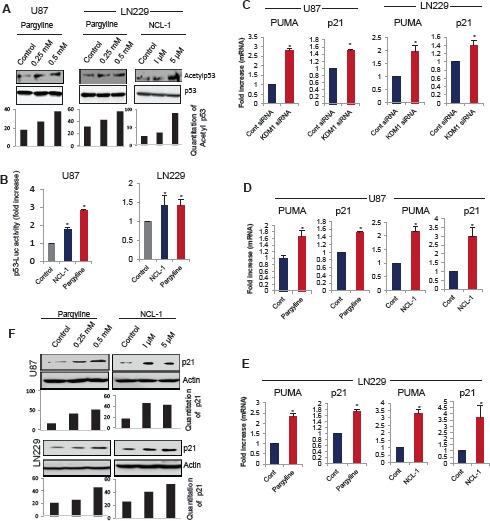
KDM1 inhibition enhanced p53 functions and its target gene activation (A) Whole cell lysates were isolated from vehicle-, pargyline- or NCL-1 treated U87 and LN229 cells and subjected to Western blot analysis with p53 and acetyl-p53^382^ antibodies (upper panel). Band intensity of acetyl p53 was quantitated by densitometry and normalized to total p53. (B) U87 and LN229 cells were transiently transfected with the p53-Luc reporter and 24 h post transfection, cells were treated with vehicle, pargyline or NCL-1. The reporter gene activity was measured after 24 h. All data presented are the mean of 3 independent experiments with mean ± SEM. *, *p*< 0.05, *t* test. (C) U87 and LN229 cells were transfected with control siRNA or KDM1 siRNA and 72 h after transfection, RNA was isolated and subjected to RT-qPCR using the primers specific for p53 target genes p21 and PUMA. All data presented are the mean ± SEM. *, *p*< 0.05, *t* test. Total RNA was isolated from vehicle-, pargyline or NCL-1 treated U87 (D) and LN229 (E) cells and subjected to RT-qPCR using the primers specific for p21 and PUMA. All data presented are the mean ± SEM. *, *p*< 0.05, *t* test. (F) Whole cell lysates were isolated from vehicle-, pargyline- or NCL-1-treated U87 and LN229 cells and subjected Western blot analysis with p21 antibodies. β-actin used as an internal control. Band intensity of p21was quantitated by densitometry and normalized to total actin.

Recent studies suggest that in addition to modulation of histone substrates, KDM1 associates with p53 and regulates its function by demethylation [12] and that the interplay between p53 methylation and acetylation provide mechanisms for triggering a rapid increase in p53 transcriptional activity. Because inhibition of KDM1 decreased glioma proliferation, we examined whether pharmacological inhibition of KDM1 enhanced acetylation of p53^382^, a known modification that activates the p53 stability and functions. Pargyline and NCL-1 treatments substantially increased the levels of acetyl-p53^382^ in both U87 and LN229 glioma cells (Fig. 3A). The total p53 levels were not altered after KDM1 inhibition. We next validated the activation of p53 by KDM1 inhibition using p53 reporter gene assays. As shown in Fig 3B, both the pargyline and NCL-1 treatments significantly increased the p53 reporter activity in both U87 and LN229 glioma cell lines. p53 induces cell cycle arrest and apoptosis by activating the transcription of its target genes p21 and PUMA, respectively. RT-qPCR analysis showed that knockdown of KDM1 significantly increased the mRNA levels of p21 and PUMA in both U87 and LN229 cells (Fig 3C). Similarly, treatment with either pargyline or NCL-1 also significantly increased the p21 and PUMA expression in both U87 and LN229 cell lines (Fig. 3D,E). Accordingly, Western blot analysis showed that treatment with either pargyline or NCL-1 significantly enhanced the p21 levels in both U87 and LN229 cells (Fig. 3F).

### KDM1 inhibition leads to reduced stem-like glioma cell proliferation

**Figure 4:**
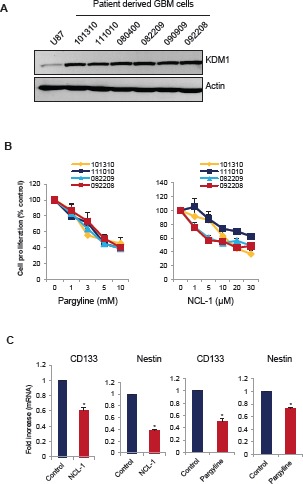
KDM1 inhibitors reduced proliferation of SLGCs (A) Whole cell lysates from various primary GBM cells were subjected to Western blot analysis with KDM1 antibody. β-actin used as an internal control. (B) Primary GBM cells (#101310, 111010, 082209, and 092208) were seeded in 96-well plates and treated with varying concentrations of pargyline or NCL-1 for 72 h and subjected to MTS assay as described in the methods. (C) Primary GBM cells (#111010) were treated with vehicle, pargyline or NCL-1 for 7 days and total RNA was isolated and subjected to RT-qPCR with primers specific for CD133 and nestin. All data presented are the mean ± SEM. *, *p*< 0.05, *t* test.

Recent studies indicated that KDM1 regulates neural stem cell proliferation [21], and that metastatic behaviour and resistance to conventional radiation and chemotherapy of glioblastomas is attributed to the presence of stem-like glioma cells (SLGCs) [22;23]. To test whether inhibition of activity of KDM1 reduces SLGCs proliferation, we treated the SLGCs derived from primary GBM samples with pargyline or NCL-1. All the SLGCs tested have elevated levels of KDM1 (Fig. 4A). Pargyline and NCL-1 dose-dependently reduced the proliferation of SLGCs (Fig. 4B). We then studied the expression of neural stem markers such as Nestin and CD133 after treatment with either pargyline or NCL-1. The expression levels of Nestin and CD133 were significantly downregualted in SLGCs after the pargyline and NCL-1 treatments (Fig. 4C). These results further confirm that KDM1 is required for maintaining stemness of SLGCs.

### Pargyline treatment inhibits the glioma xenograft tumor growth and induces apoptosis

**Figure 5:**
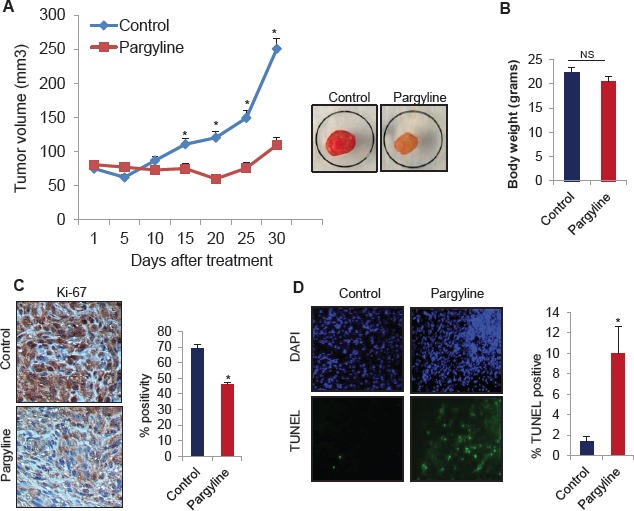
Pargyline treatment reduced subcutaneous glioma xenograft tumor growth and induced apoptosis *in vivo* (A) NOD/SCID mice were subcutaneously implanted with 1 × 10^6^ U87 cells. After tumors reached a measurable size, mice were treated daily with vehicle or pargyline (100 mg/kg/ bodyweight) for a period of 30 days. Tumor size was measured with calipers every 5 days. A representative picture of a tumor is shown as an inset. (B) Body weights of both vehicle- and pargyline-treated mice were measured weekly. Column, mean body weights. (C) Ki-67 staining in control- and pargyline-treated tumors. Representative images are depicted (left panel). * *P* <0.05. Ki-67 labelling was quantified as the mean Ki-67 percentage based on at least 10 randomly selected high-power microscope fields per group (right panel) (D) TUNEL staining for apoptosis in control- and pargyline-treated tumors. Representative images are depicted (left panel). TUNEL labelling was quantified as the mean TUNEL labelling percentage based on at least 10 randomly selected high-power microscope fields per group (right panel).

To study the therapeutic effect of KDM1 inhibition on the growth of gliomas *in vivo*, we used a nude mice-based subcutaneous glioma xenograft model. NOD/SCID mice were subcutaneously implanted with 1 × 10^6^ U87 glioma cells in their flank regions. When the tumors reached measurable size, the mice were given either pargyline or PBS intraperitonially. Tumor volumes were measured every 5 days and mice were euthanized 30 days after treatment. Compared to PBS, pargyline treatment significantly reduced the tumor growth (Fig.5A). No toxicities were observed in pargyline treatment group as determined by behavioural changes, such as eating habits and mobility, and mouse weights were not significantly different between the control and pargyline-treated groups (Fig. 5B). The expression of Ki-67 (proliferation marker) was significantly lower in the pargyline-treated tumors than in the control tumors (Fig. 5C). TUNEL analysis showed that the number of apoptotic cells was significantly higher in the pargyline-treated tumors than in the control tumors (Fig. 5D). These results suggest that pargyline-mediated reduction in xenograft tumor growth involves both a reduction of proliferation and an induction of apoptosis.

### Pargyline treatment increases the global H3K4 methylation and H3K9 acetylation and reduces H3K9 methylation marks in xenograft tumors

**Figure 6:**
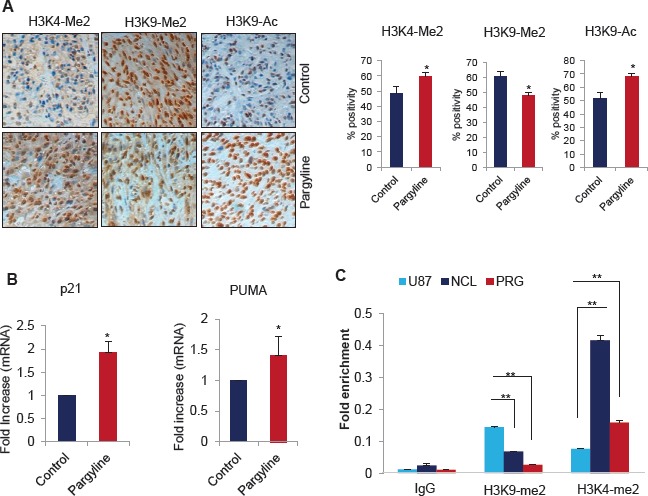
Pargyline treatment increased the global H3K4 methylation and H3K9 acetylation marks and reduced the H3K9 methylation in xenograft tumors (A) IHC analysis of H3K4-me2, H3K9-me2 and H3K9-Ac marks was done on xenograft tumors that were treated with vehicle or pargyline (Left panel). Quantitation was done as described in the methods section. All data presented are the mean ± SEM. *, *p*< 0.05. (Right panel). (B) Total RNA was isolated from control or pargyline-treated tumors and subjected to RT-qPCR using the primers specific for p21 and PUMA. All data presented are the mean ± SEM. *, *p*< 0.05. (C) U87 cells were treated with pargyline or NCL-1 and ChIP analysis was performed using H3K4-me2 and H3K9-me2 antibodies and the status of epigenetic modification was analyzed using real-time PCR with p21 promoter-specific primers.

To examine whether pargyline blockage of the KDM1 demethylase function affects tumor growth via epigenetic modifications, we determined the status of global H3K4 and H3K9 methylation and H3K9 acetylation marks in xenograft tumors. Immunohistochemical analysis showed that global H3K4 methylation and H3K9 acetylation were significantly greater in pargyline-treated tumors than in control tumors (Fig. 6A). We also observed that H3K9 methylation was significantly reduced in pargyline-treated tumors than in control tumors (Fig. 6A). To examine whether the effects reflect the expression of p53 target genes involved in the repression of growth, we analyzed mRNA expression of p21 and PUMA by using total RNA isolated from xenograft tumors. The expression levels of p21 and PUMA were significantly higher in pargyline-treated xenograft tumors than in control tumors (Fig. 6B). To test whether pargyline treatment promoted the alteration in epigenetic marks at the p53 response element on p21 promoter, we examined the status of the H3K4-me2 and H3K9-me2 marks after treating U87 cells with pargyline. A substantial increase was found in the H3K4-me2 and reduction on H3K9-me2 at the p21 promoters (Fig. 6C). These studies suggest that blockage of the KDM1 axis via pargyline has potential to decrease glioma proliferation *in vivo* and that pargyline promotes tumor suppressive effects probably by epigenetic modification at promoters of p53 target genes.

## DISCUSSION

Glioma development is a multistep process that involves various genetic and epigenetic alterations [24]. Unlike genetic alterations, epigenetic changes are reversible and thus, targeting epigenetic changes represents a promising therapeutic approach. KDM1 regulates gene expression programs by changing the epigenetic histone marks in gene promoters [10]. In this study, we tested the hypothesis that deregulation of KDM1 promotes glioma progression and tested the therapeutic effect of targeting KDM1 axis using *in vitro* and *in vivo* methods. The results using tumor tissue microarray studies revealed that KDM1 is overexpressed in glioma tissues and its expression positively correlates with histological malignancy. Further, our results demonstrated that pharmacological inhibition of KDM1 activity or knockdown of KDM1 expression decreases the proliferation of glioma cell lines by modulating the p53 pathway.

The transcriptional factor p53 functions as a guardian of the genome by modulating appropriate responses to stress. The function of p53 is tightly regulated by various post-translational modifications including acetylation, and methylation. KDM1 demethylates p53K370me2, which prevents its interaction with the coactivator 53BP1, thereby blocking the p53 transcriptional activation and induction of p53-mediated apoptosis [12]. The acetylation of lysines at the C-terminus of p53 protects p53 from ubiquitination, and acetylation at lys382 increases the DNA binding activity of p53 [25] and promotes its interaction with other transcriptional factors [26]. In the present study, we observed that the KDM1 inhibitors pargyline and NCL-1 increase the levels of acetyl-p53^382^ and increase p53 reporter gene activation. Further, KDM1 inhibition increased induction of the p53 target genes p21 and PUMA. These findings suggest that the blockage of KDM1 functions via pharmacological inhibition may provide a favourable environment for the activation of p53 and its target genes, leading to suppression of gliomas.

KDM1 plays a key role in many physiological functions, and recent studies suggested that KDM1 is often overexpressed in various cancers [27]. KDM1 knockdown reduces proliferation by affecting expression of several genes involved in proliferation including p21, CCNA2, and ERBB2, which arrests the cell cycle at G1/S phase [15]. G0/G1 phase [28] and G2/M phase [29]. In this study, we observed that inhibition of activity or knockdown of KDM1 expression reduced proliferation and colony formation of glioma cell lines *in vitro* and reduce tumor growth *in vivo*. Recent TCGA studies revealed TP53 mutations in ~35% gliomas, suggesting an alternate mechanism of WTp53 inactivation occurs in the remaining ~65% of gliomas. Since KDM1 expression is upregulated in gliomas, because KDM1-mediated demethylation reduces p53 transactivation functions, blocking the KDM1 axis could have therapeutic implications for reducing glioma proliferation in WTp53 expressing gliomas.

Recent studies implicate KDM1 as a key player in the maintenance of pluripotency and high levels of KDM1 are needed to maintain the undifferentiated state of human embryonic stem cells [28]. Knockdown of KDM1 or inhibition of its enzymatic activity results in the selective inhibition of pluripotent stem cells proliferation but a lesser effect has been seen on non-pluripotent cancer or normal somatic cells [30]. Recent studies also highlighted the importance of KDM1 in the regulation of neural stem cell proliferation and differentiation. In neural stem cells KDM1 is recruited to the TLX target genes to repress their expression, which regulates the proliferation of neural stem cells and it has also been showed that knockdown of KDM1 or treatment with the KDM1 inhibitors pargyline and tranylcypromine reduced the proliferation of hippocampus neural progenitor cells [21]. Inhibition of KDM1 is also shown to reactivate the ATRA-induced differentiation in acute myeloid leukaemia [31]. In this study, we demonstrated that KDM1 is overexpressed in SLGCs and treatment with KDM1 inhibitors reduces the proliferation of SLGCs. Further KDM1 inhibitors decreased the expression of stemness genes in SLGCs.

KDM1 is a target for monoamine oxidase inhibitors including pargyline and tranylcypromine, which are currently used as antidepressants and are shown to inhibit KDM1 activity. In our study, we used pargyline (trade name: Eutonyl, Supirdyl, Eutron), an FDA approved drug to treat depression and vascular hypertension. Pargyline had significant growth inhibitory properties *in vitro.* A recent study using *in vitro* assays found synergistic apoptosis occurred when the combination of KDM1 and HDAC inhibitors was used [32], also supporting our findings. In our study, we further extended these findings and tested the therapeutic potential of the KDM1 inhibitor pargyline *in vivo* by using a xenograft model. Pargyline treatment reduced glioma growth, increased levels of H3K4-me2 and H3K9-Ac and reduced levels of H3K9-me2, and these changes correlated with increased apoptosis. This proof of principle *in vivo* study demonstrated the significance of the KDM1 axis in curbing glioma progression. However, the extended period use of a high concentration of pargyline may cause side effects. To overcome this, we are currently developing better inhibitors of KDM1 that work efficiently at lower doses with high specificity. We recently developed the compound NCL-1 that showed significant inhibitory activity on glioma cells in~20 μM range. Using the glioma model cells, we demonstrated that NCL-1 treatment promoted p53 functions as well as modulated optimal epigenetic changes, leading to the activation p53 target genes. Future studies are clearly needed to test its *in vivo* potential, safety and utility in combinatorial therapy.

In summary, our study findings establish that deregulation of KDM1 expression occurs during glioma progression and provides the first *in vivo* evidence demonstrating the KDM1 axis as a potential therapeutic target for gliomas. Inhibition of KDM1 alone or in combination with other epigenetic-modifying agents may be a potential tool in the therapeutic intervention of gliomas.

## MATERIALS AND METHODS

### Cell lines and reagents

Human glioma cell lines U87, T98G, LN229, U138, M059J, M059K and the breast cancer cell line MCF7 were obtained from the American Type Culture Collection (ATCC) and were passaged in our laboratory for less than six months. Glioma cell lines were maintained in DMEM medium, and MCF7 cells were maintained in RPMI-1640 medium supplemented with 10% FBS (Hyclone Laboratories Ltd, Logan, UT). Patient-derived primary glioblastoma cells 101310, 111010, 080400, 082209, 090909 and 092208 were generated from surgically resected tumors as described [16] and propagated in neurobasal medium supplemented with B27 serum-free supplement, EGF (20 ng/ml), bFGF (20 ng/ml), LIF (20 ng/ml) and heparin (5 μg/ml). KDM1-specific siRNA were obtained from Thermo Scientific (Waltham, MA). The KDM1, H3K4-me2, H3K9-me2 and H3K9-Ac antibodies were obtained from Millipore (Billerica, MA), and p53, acetyl-p53, p21 and PUMA antibodies were purchased from Cell Signalling Technology (Danvers, MA). Ki67 was obtained from Dako (Carpinteria, CA). Pargyline, β-actin and all secondary antibodies were purchased from Sigma Chemical Co (St. Louis, MO). N-[(1S)-3-[3-(trans-2-Aminocyclopropyl)phenoxy]-1-(benzylcarbamoyl)propyl]benzamide (NCL-1) was synthesized as previously described [17].

### Cell lysis and Western blotting

Whole cell lysates were prepared from glioma cells in modified RIPA buffer (150 mM NaCl, 50 mM Tris-HCl, 50 mM NaF, 5 mM EDTA, 0.5% [wt/vol] sodium deoxycholate and 1% Triton X-100) containing phosphatase and protease inhibitors. Lysates were run on SDS-PAGE followed by Western blotting using indicated antibodies and developed using the ECL methodology.

### Cell proliferation and clonogenic assays

Cell proliferation rates were measured by using MTT Cell Viability Assay in 96-well microplates. Glioma cells were seeded in 96-well plates (2 × 10^3^ cells/well) in DMEM medium containing 10% serum. After an overnight incubation, cells were treated with varying concentrations of pargyline or NCL-1 for 72 h and growth inhibition was determined by using traditional MTT assays. For the primary GBM cells, single-cell suspensions were plated in 96-well plates (1 × 10^3^ cells/well), and pargyline- or NCL-1-mediated growth inhibition was determined by using traditional MTS assays. For the clonogenic assays, U87 and LN229 cells (500 cells/well) were seeded in 6-well plates. After an overnight incubation, cells were treated with pargyline (3 mM) or NCL-1 (10 μM) for 72 h. Then cells were washed with PBS and allowed to grow for an additional 7 days. The cells were then fixed in ice cold methanol and stained with 0.5% crystal violet solution to visualize the colonies. Colonies that contain ≥ 50 cells were counted.

### Quantitative RT-PCR analysis

Total RNA was isolated from glioma cells and xenograft tumor tissues using Trizol Reagent (Invitrogen, Carlsbad, CA) according to the manufacturer's instructions. Reverse transcription (RT) reactions were performed by using the Superscript III reagent kit (Invitrogen, Carlsbad, CA). Real-time PCR was done by using a Cepheid Smart cycler II (Sunnyvale, CA) with specific real-time PCR primers for following genes: p21: (F) CTGGAGACTCTCAGGGTCGAAA and (R) GATTAGGGCTTCCTCTTGGAGAA; PUMA: (F) ATGCCTGCCTCACCTTCATC and (R) TCACACGTCGCTCTCTCTAAACC; Nestin: (F) AGACACCTGTGCCAGCCTTTC and (R) CTGCTGCAAGCTGCTTACCAC; CD133: (F) GAACAAGTTTACAGTGACTGC and (R) TGCGTTGAAGTATCTTTGACG; and Actin: (F) GTGGGCATGGGTCAGAAG and (R) TCCATCACGATGCCAGTG. Results were normalized to the β-actin transcript levels and the difference in fold expression was calculated by using delta-delta-CT method.

### Reporter gene assays

Reporter gene assays were performed as described [18]. Briefly, glioma cell lines U87 and LN229 were seeded in 6-well plates. After overnight incubation, the cells were transfected with p53-Luc plasmid using fugene for 6 h. Then, 24 h after transfection, cells were treated with vehicle, pargyline or NCL-1 for an additional 24 h. Each transfection was carried out in triplicate and normalized with the β-gal activity and total protein concentration. Luciferase activity was measured by using the luciferase assay system (Promega, Madison, WI). Luciferase activity was expressed as percent of relative light units versus untreated transfected cells.

### Tissue microarrays and immunohistochemistry

The tissue microarrays (TMAs) were obtained from US BioMax (Rockville, MD). Each TMA comprised 0.6-mm cores taken from paraffin-embedded specimens that represent a total of 192 glioma tissues and 8 each of adjacent normal tissue and normal brain tissues. Immunohistochemical analysis was performed using Allred scoring methodology as described in our earlier publication [18]. Allred score takes into consideration the proportion of positive cells (scored on a scale of 0-5) and staining intensity (scored on a scale of 0-3). The proportion of positive stained cells was rated as 1 = between 0% and 1% positive, 2 = between 1% and 10%, 3 = between 10% and 33%, 4 = between 33% and 66%, and 5 = between 66% and 100%. The preparation of negative controls was accomplished by replacing the primary antibody with control rabbit IgG. The sections were scored by 3 evaluators blinded to the patient's clinical status. Xenograft tumor sections were incubated overnight with Ki-67, H3K4-Me2, H3K9-me2 and H3K9-Ac primary antibodies and positivity was visualized by using the DAB substrate and counterstained with haematoxylin (Vector Lab, Inc. Burlingame, CA). The proliferative index was calculated as the percentage of Ki-67-positive cells in 10 randomly selected microscopic fields at 100X per slide. TUNEL analysis was done by using the *In situ* Cell Death Detection Kit (Roche, Indianapolis, IN) as per the manufacturer's protocol and 10 randomly selected microscopic fields in each group were used to calculate the relative ratio of TUNEL-positive cells.

### Chromatin immunoprecipitation assay

U87 cells were seeded in 100 mm plates and after overnight incubation cells were treated with vehicle or pargyline. Then, cells were cross-linked using 1% formaldehyde, and the chromatin was subjected to immunoprecipitation using H3K4-me2 and H3K9-me2 antibodies. Isotype-specific IgG was used as a control. Extracted DNA was dissolved in TE buffer and subjected to real-time PCR using p21 specific p53RE primers. p21 (-1391) F-CTGTCCTCCCCGAGGTCA, p21 (-1391) R- ACATCTCAGGCTGCTCAGAGTCT.

### Xenograft studies

All animal experiments were performed after obtaining UTHSCSA-IACUC approval. The animals were housed in accordance with UTHSCSA institution's protocol for animal experiments. For xenograft tumor assays, 1 × 10^6^ U87 cells were mixed with an equal volume of matrigel and implanted subcutaneously into the flanks of 6-week-old female NOD/SCID mice as described [18]. Once the tumors reached measurable size, mice were divided into control and treatment groups. The control group received PBS, and the treatment group received pargyline (100 mg/kg in PBS) i.p. per day for a period of 30 days. Tumor volumes were measured with a caliper at 5-day intervals. After the 30th day, the mice were euthanized, and the tumors were isolated and processed for histological studies. Tumor volume was calculated by using a modified ellipsoidal formula: tumor volume = ½ (L × W2), where L is the longitudinal diameter and W is the transverse diameter [18]. Body weight was measured at weekly intervals for monitoring of the drug toxicity.

### Statistical analysis

SPSS software was used for all statistical analyses. A Student's t-test was used to assess statistical differences between control and pargyline-treated groups. The level of significance was set at P<0.05. Statistical differences among groups were analyzed with ANOVA.

### Funding

This study was supported by NIH-CA0095681, NS050730 and Cancer Center Support Grant P30CA054174
